# Subcortical brain volumetric differences related to white matter lesion volume and cognition in healthy aging

**DOI:** 10.1038/s41514-025-00234-z

**Published:** 2025-05-28

**Authors:** Hyun Song, Pradyumna K. Bharadwaj, Matthew D. Grilli, David A. Raichlen, Christian G. Habeck, Matthew J. Huentelman, Georg A. Hishaw, Theodore P. Trouard, Gene E. Alexander

**Affiliations:** 1 Department of Psychology, University of Arizona, Tucson, AZ USA; 2 Evelyn F. McKnight Brain Institute, University of Arizona, Tucson, AZ USA; 3 Department of Neurology, University of Arizona, Tucson, AZ USA; 4 Human and Evolutionary Biology Section, Department of Biological Sciences, University of Southern California, Los Angeles, CA USA; 5 Cognitive Neuroscience Division, Department of Neurology and Taub Institute, Columbia University, New York, NY USA; 6 Neurogenomics Division, The Translational Genomics Research Institute (TGen), Phoenix, AZ USA; 7 Arizona Alzheimer’s Consortium, Phoenix, AZ USA; 8 Department of Biomedical Engineering, University of Arizona, Tucson, AZ USA; 9 Department of Psychiatry, University of Arizona, Tucson, AZ USA; 10 Neuroscience and Physiological Sciences Graduate Interdisciplinary Programs, University of Arizona, Tucson, AZ USA

**Keywords:** Cognitive ageing, Neural ageing, Risk factors

## Abstract

White matter hyperintensity (WMH) lesions associated with small vessel cerebrovascular disease (CVD) are common structural neuroimaging findings in older adults. Greater global brain WMH burden related to aging has been implicated in dementia but has also been linked to brain atrophy and cognitive dysfunction in old age. We sought to investigate the regionally distributed association of global WMH lesion load with subcortical gray matter (SGM) volumes using a multivariate network analysis method in 178 community-dwelling, healthy older adults (mean age = 69.77 ± 10.22 years). We additionally applied mediation models with WMH-related subcortical volumetric differences as a mediator to evaluate a potential global WMH-related vascular risk pathway leading to cognitive aging. Global WMH burden was associated with a regionally distributed pattern of SGM atrophy involving bilateral putamen and left nucleus accumbens, with relative volume increases in bilateral caudate nucleus. Mediation analyses revealed that increasing age predicted greater WMH-SGM pattern expression, which then predicted slowed processing speed that was, in turn, associated with decrements in other age-sensitive cognitive domains of memory, executive functioning, and fine motor function. These results suggest that the multivariate WMH-SGM pattern and its association with processing speed may provide an important early indicator of age-related decrements in higher-order cognitive processes, reflecting a potential link between CVD and broader cognitive dysfunction across multiple domains in healthy aging.

## Introduction

White matter hyperintensity (WMH) lesions, commonly observed on magnetic resonance imaging (MRI) in healthy aging^1^, provide a neuroimaging marker of white matter lesion load associated with small vessel cerebrovascular disease (CVD)^2^. Increased WMH volume in old age has been implicated in cognitive impairment^3–5^ and an increased risk of Alzheimer’s disease and related dementias (ADRD)^2^. Subcortical gray matter (SGM) atrophy has been associated with WMH lesions and has been proposed as a major contributor to cognitive impairment and dementia risk in old age^6–11^. Prior work has shown ventricular enlargement, atrophy of the basal ganglia (including striatal volume reductions), and hippocampal atrophy in relation to greater WMH volume in aging^9,12–18^, which may in turn contribute to cognitive dysfunction associated with white matter lesion load^16,19^, but these relationships have yet to be fully elucidated. Investigating the relation of WMH to SGM volume differences in healthy older adults may help to further clarify how WMH lesions influence cognitive dysfunction in aging. This work could also help to identify a potential pathway linking small vessel CVD to cognitive aging, while providing a possible early indicator for dementia risk.

While previous research has reported volume reductions in regions often impacted in aging and ADRD, the focus has been largely restricted to cortical brain regions^14,16,20,21^. Even studies examining WMH-related subcortical brain atrophy in aging have primarily focused on selective brain regions, particularly the hippocampus^16,17,22^, or individual regions in isolation^8,11,23,24^. Less attention has been directed toward other key subcortical brain areas that have been implicated in aging and dementia, such as the striatum^8,11,23,24^. Although it has been increasingly recognized that between-person differences in the volume of a brain region often covary with individual differences in the volume of other brain structures^20,25^, studies have yet to consider covariance patterns across subcortical brain structures that reflect the regionally distributed associations between WMH lesion load and multiple SGM volumes.

Unlike traditional univariate approaches that assess differences in brain regions individually, multivariate analysis methods in structural neuroimaging studies can be used to characterize regional patterns or networks of brain structure. As a multivariate method, the Scaled Subprofile Model (SSM) is a network covariance analysis technique that can detect distributed patterns of structural brain volumes related to inter-individual differences in clinical and demographic characteristics or other relevant brain measures without requiring conservative multiple comparison correction^26^. This technique applies a principal component-based approach to identify multivariate patterns of variance shared across individuals that reflect relative differences over multiple brain regions^27,28^. The SSM provides a set of regional pattern weights and expression scores that reflect the degree to which each participant expresses the observed pattern, making it capable of addressing sources of variability in neuroimaging datasets related to other variables of interest^26^.

Applications of this analytic method to structural brain imaging data have shown sensitivity in identifying network patterns of gray matter volume reductions in human studies of healthy aging and vascular risk burden^21,29,30^, and in non-human primate and rodent models of aging^31,32^. Moreover, the SSM analysis has been shown to effectively capture subtle regional volumetric patterns that link different measures of brain structure^21,33^. As such, applying the SSM provides a way to identify a multimodal neuroimaging covariance pattern of SGM volume differences related to WMH lesion load and to examine its linkage to cognitive function in aging.

Previous research on cognitive dysfunction related to WMH burden in aging has reported mixed findings across studies regarding associations between WMH lesions and age-sensitive cognitive domains, including verbal memory, executive functioning, and fine motor skills^3–5,34,35^. However, slowed information processing speed, commonly observed in vascular cognitive impairment^36^, has been suggested to be preferentially related to white matter lesion load^2,3^. Additionally, cumulative evidence has demonstrated that a large portion of observed cross-sectional age-related differences in higher-order cognitive functions, such as verbal memory and executive functions, can be attributed to slowing of processing speed^37,38^. These findings, taken together, suggest that slowed processing speed may play a central role as an important link in mediating associations between WMH lesions and deficits in other higher-order cognitive domains preferentially affected by aging.

In the present study, we sought to identify the regionally distributed pattern of volume reductions in SGM associated with global WMH burden in healthy aging using the SSM^26^. We hypothesized that subcortical brain regions previously shown to be associated with WMH lesion load, including hippocampus, nucleus accumbens, caudate, and putamen, would demonstrate volume reductions as part of a WMH-related regional covariance pattern of SGM volumes. We additionally applied a series of mediation models to evaluate a WMH-related vascular risk pathway contributing to cognitive aging. We further hypothesized that greater expression of the WMH-SGM network pattern related to aging would be associated with diminished processing speed, which, in turn, would be associated with decrements in other cognitive abilities typically affected by aging.

## Results

### Network covariance pattern

**Fig. 1:**
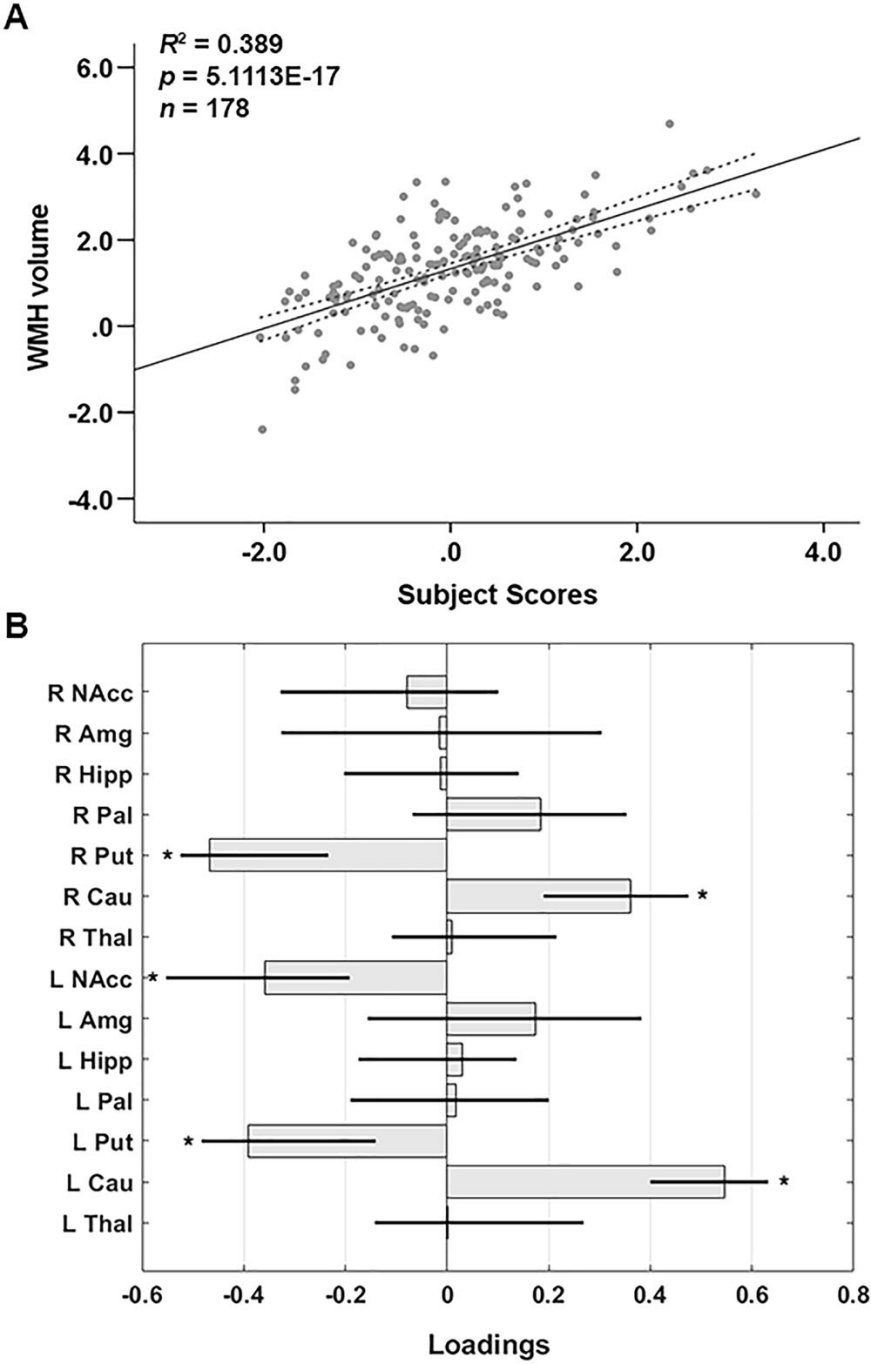
WMH-related SGM network pattern in healthy older adults. **A** Network participant scores and WMH. The participant scores of the WMH-related SGM pattern were derived from the first seven SSM components. Total WMH volume was log-transformed. The scatter plot shows that greater total WMH volume was associated with greater expression of the WMH-related SGM network SSM pattern. The dotted lines indicate 95% CIs. Adjusted *R*^2^ value is indicated. **B** WMH-related SSM loadings for the network pattern of the bilateral volumes of subcortical brain structures. Loadings are directional weights that reflect the associations between volume of each subcortical brain structure and WMH lesions in the context of the identified linearly combined SSM network pattern. Negative loadings represent regions showing WMH-related decreases in SGM volume, while positive loadings represent regions showing relative SGM volume increases related to WMH lesion volume. The gray bars represent point estimates for the SSM loadings, black lines represent bootstrap 95% CIs with 10,000 iterations, and asterisks indicate statistically significant SGM regions contributing to the SSM network pattern. Amg amygdala, Cau caudate, CI confidence interval, Hipp hippocampus, L left, NAcc nucleus accumbens, Pal pallidum, Put putamen, R right, SGM subcortical gray matter, SSM Scaled Subprofile Model, Thal thalamus, WMH white matter hyperintensity.

A multivariate SSM network analysis identified a covariance pattern reflecting how global WMH volume was associated with regional differences in SGM volumes. A linear combination of the first seven SSM components that represent a regional covariance pattern of SGM volume associated with global WMH burden was selected based on the lowest Bayesian information criterion (BIC) value. This regression model accounted for 38.9% of the variance in total WMH volume in the sample (*F*_(7,170)_ = 17.10, *p* = 5.1113E-17; Fig. 1A). The WMH-related SSM pattern that reflects a linear combination of the seven components was characterized by reduced volumes of bilateral putamen and left nucleus accumbens, with relatively greater volumes of the caudate nucleus bilaterally (Fig. 1B).

### Age predicting cognitive functioning through the WMH-SGM pattern

We then examined whether the association of age with cognitive functioning was mediated through the WMH-SGM network pattern, testing measures of age-sensitive cognitive domains separately as an outcome variable. The simple mediation models revealed that the association between age and processing speed was mediated through the WMH-SGM network pattern (Effect = 0.115, *SE* = 0.043, 95% confidence intervals [CI] [0.035; 0.203], with a diminished, but still significant direct effect of age on processing speed performance after adjusting for total intracranial volume (TIV), sex, years of education, apolipoprotein E (APOE) ε4 carrier status, hypertension status, and the time interval between MRI scans and neuropsychological assessment (Table 1). Specifically, increasing age was associated with greater pattern expression (*β* = 0.492, *p* < 0.0001), which was then associated with poorer (i.e., slowed) performance on the Trail Making Test, Part A (TMT-A; *β* = 0.234, *p* = 0.0054).

**Table 1:** Summary of simple mediation models of age predicting cognitive functioning through the WMH-related SGM pattern

Cognitive measures	Indirect effects			Direct effects	Total effects
	Effect	*SE*	95% CI	Effect	Effect
SRT CLTR	0.025	0.040	−0.052, 0.105	−0.497^***^	−0.472^***^
TMT-B^a^	−0.060	0.044	−0.152, 0.022	0.234^**^	0.174^*^
WAIS-IV LNS	−0.030	0.044	−0.120, 0.056	−0.416^***^	−0.447^***^
SCWT	−0.008	0.040	−0.089, 0.070	−0.423^***^	−0.430^***^
TMT-A^b^	**0.115**	0.043	0.035, 0.203	0.262^**^	0.377^***^
GPT^b^	0.058	0.034	−0.003, 0.131	0.639^***^	0.697^***^

Effect and *SE* represent standardized coefficients and standard error, respectively, while adjusting for TIV, sex, years of education, APOE ε4 status, hypertension status, and the time interval between MRI scans and neuropsychological tests. CIs indicate statistical significance when they do not contain zero. The indirect effects that remained significant after additionally adjusting for high cholesterol status, smoking history, BMI, and VO_2_max (Effect = 0.091, *SE* = 0.039, 95% CI [0.019; 0.173]) are bolded.

*APOE* apolipoprotein E, *BMI* body mass index, *CI* confidence interval, *CLTR* consistent long-term retrieval, *GPT* Grooved Pegboard Test, *LNS* letter number sequencing, *MRI* magnetic resonance imaging, *SCWT* Stroop Color-Word Interference Test, *SGM* subcortical gray matter, *SRT* Buschke Selective Reminding Test, *TIV* total intracranial volume, *TMT* Trail Making Test, *VO*_2_*max* volume of maximal oxygen consumption, *WAIS-IV* Wechsler Adult Intelligence Scale–Fourth Edition, *WMH* white matter hyperintensity.

^a^ Standardized residual value after statistically removing the processing speed performance on TMT-A with raw scores of the TMT.

^b^ Log-transformed value.

^*^ *p* < 0.05, ^**^ *p* < 0.01, ^***^ *p* < 0.001.

With the same covariates, however, there were no significant mediation effects of the WMH-SGM network pattern in the association between age and verbal memory on the consistent long-term retrieval (CLTR) score from the Buschke Selective Reminding Test (SRT) (Effect = 0.025, *SE* = 0.040, 95% CI [−0.052; 0.105]) and fine motor function on the Grooved Pegboard Test (GPT) with the dominant hand (Effect = 0.058, *SE* = 0.034, 95% CI [−0.003; 0.131]; Table 1). Similarly, no significant indirect effects of the network pattern were observed for three components of executive functioning (Effect = −0.060, *SE* = 0.044, 95% CI [−0.152; 0.022] for shifting on the Trail Making Test, Part B [TMT-B]; Effect = −0.030, *SE* = 0.044, 95% CI [-0.120; 0.056] for working memory on the Letter-Number Sequencing [LNS] from the Wechsler Adult Intelligence Scale–Fourth Edition [WAIS-IV]; and Effect = −0.008, *SE* = 0.040, 95% CI [−0.089; 0.070] for inhibition on the Stroop Color-Word Interference Test [SCWT]) while controlling for the covariates listed above (Table 1).

Additionally adjusting for other vascular health risk factors of smoking history, maximal oxygen uptake (VO_2_max), body mass index (BMI), and high cholesterol status did not appreciably diminish the mediation effect for processing speed (Effect = 0.091, *SE* = 0.039, 95% CI [0.019; 0.173]). In contrast, a similar pattern of results was observed for the other cognitive domains: no significant mediation effects for memory (Effect = 0.031, *SE* = 0.034, 95% CI [−0.030; 0.103]), fine motor function (Effect = 0.052, *SE* = 0.031, 95% CI [−0.001; 0.119]), and executive functioning (Effect = −0.054, *SE* = 0.040, 95% CI [−0.146; 0.012] for shifting; Effect = −0.011, *SE* = 0.035, 95% CI [−0.078; 0.061] for working memory; Effect = 0.002, *SE* = 0.036, 95% CI [−0.068; 0.077] for inhibition).

### Age predicting cognitive functioning sequentially through the WMH-SGM pattern and processing speed

**Fig. 2:**
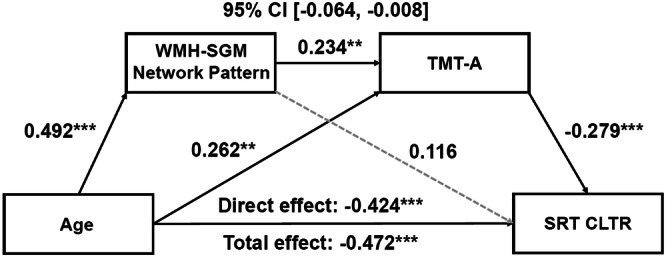
Serial mediation models of the WMH-related SGM network pattern and processing speed sequentially mediating the association between age and memory. Boxes and paths indicate hypothesized variables and their associations. Black solid lines indicate statistically significant paths, while gray dotted lines indicate non-significant paths. Standardized path coefficients with TIV, sex, years of education, APOE **ε**4 status, hypertension status, and the time interval between MRI scans and neuropsychological test administration as covariates are presented. In this model, zero does not lie within the 95% CIs, indicating a significant mediation effect. This sequential mediation effect remained significant after additionally adjusting for high cholesterol status, smoking history, BMI, and VO_2_max, and further depressed mood. APOE apolipoprotein E, BMI body mass index, CI confidence interval, CLTR consistent long-term retrieval, MRI magnetic resonance imaging, SGM subcortical gray matter, SRT Buschke Selective Reminding Test, TIV total intracranial volume, TMT Trail Making Test, VO_2_max volume of maximal oxygen consumption, WMH white matter hyperintensity. ^*^p < 0.05, ^**^p < 0.01, ^***^p < 0.001.

Subsequently, we tested whether the WMH-SGM pattern influenced other age-sensitive cognitive domains through its impact on processing speed with the same covariates listed above. The serial mediation models revealed that the relationship between age and verbal memory was mediated sequentially through the WMH-related SGM pattern and processing speed (Effect = −0.032, *SE* = 0.014, 95% CI [−0.064; −0.008]; Fig. 2), in addition to significant direct age effects.

**Fig. 3:**
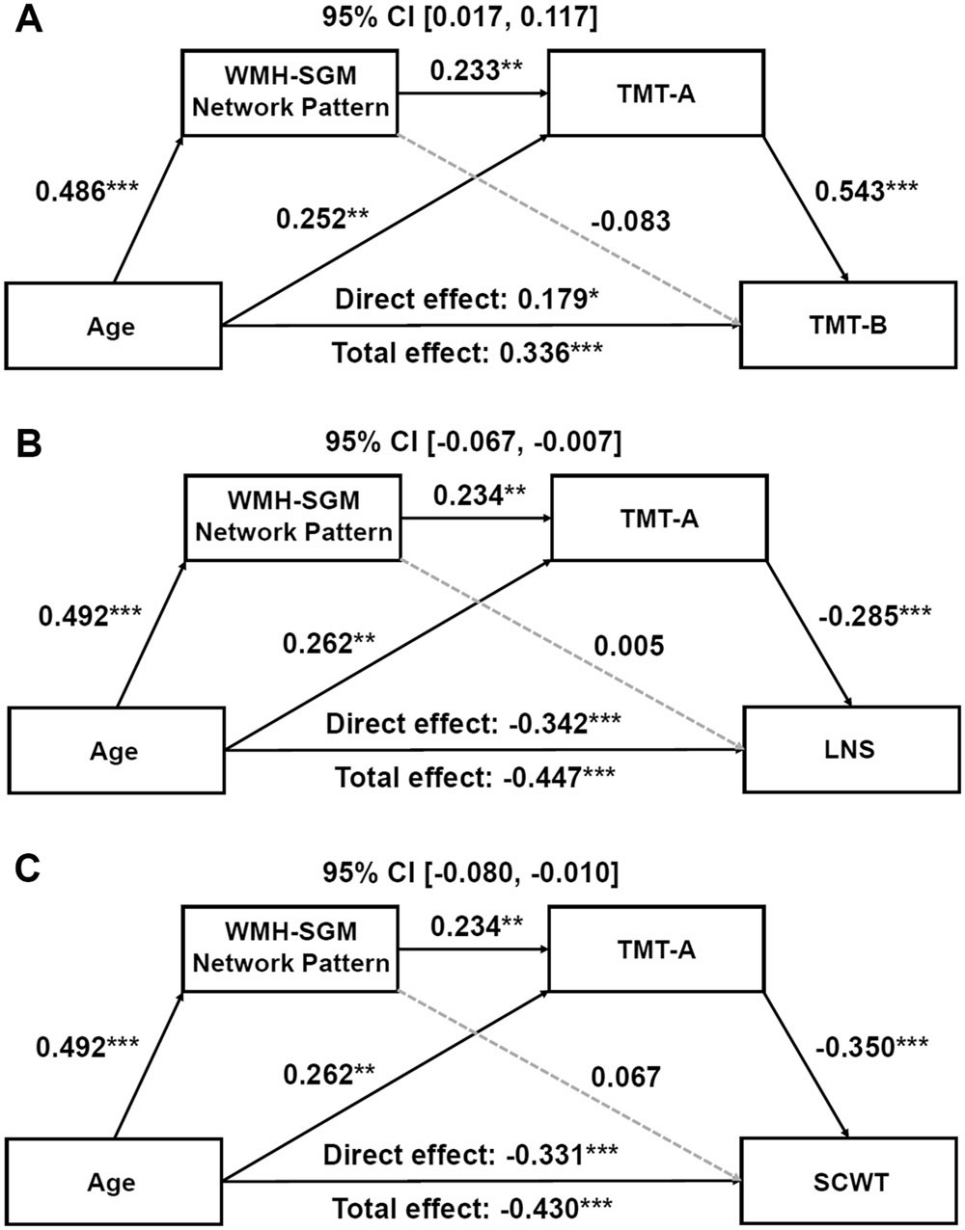
Serial mediation models of the WMH-related SGM network pattern and processing speed sequentially mediating the association between age and executive functioning. **A** TMT-B, **B** WAIS-IV LNS, and **C** SCWT. Boxes and paths indicate hypothesized variables and their associations. Black solid lines indicate statistically significant paths, while gray dotted lines indicate non-significant paths. Standardized path coefficients with TIV, sex, years of education, APOE **ε**4 status, hypertension status, and the time interval between MRI scans and neuropsychological test administration as covariates are presented. In these models, zero does not lie within the 95% CIs, indicating significant serial mediation effects. These mediation effects remained significant after additionally adjusting for high cholesterol status, smoking history, BMI, and VO_2_max, and further depressed mood. TMT-A and TMT-B were log-transformed. APOE apolipoprotein E, BMI body mass index, CI confidence interval, LNS letter number sequencing, MRI magnetic resonance imaging, SCWT Stroop Color-Word Interference Test, SGM subcortical gray matter, TIV total intracranial volume, TMT Trail Making Test, VO_2_max volume of maximal oxygen consumption, WAIS-IV Wechsler Adult Intelligence Scale–Fourth Edition, WMH white matter hyperintensity. ^*^p < 0.05, ^**^p < 0.01, ^***^p < 0.001.

**Fig. 4:**
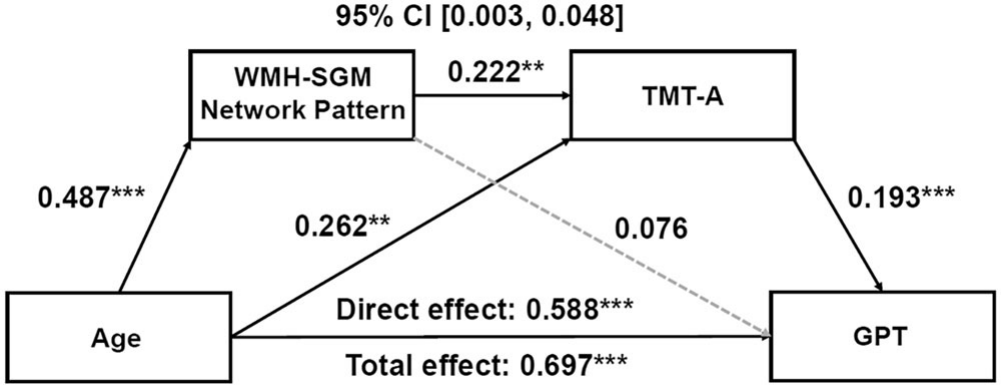
Serial mediation models of the WMH-related SGM network pattern and processing speed sequentially mediating the association between age and fine motor function. Boxes and paths indicate hypothesized variables and their associations. Black solid lines indicate statistically significant paths, while gray dotted lines indicate non-significant paths. Standardized path coefficients with TIV, sex, years of education, APOE **ε**4 status, hypertension status, and the time interval between MRI scans and neuropsychological test administration as covariates are presented. In this model, zero does not lie within the 95% CIs, indicating a significant mediation effect. This sequential mediation effect remained significant after additionally adjusting for high cholesterol status, smoking history, BMI, and VO_2_max, and further depressed mood. APOE apolipoprotein E, BMI body mass index, CI confidence interval, GPT Grooved Pegboard Test, MRI magnetic resonance imaging, SGM subcortical gray matter, TIV total intracranial volume, TMT Trail Making Test, VO_2_max volume of maximal oxygen consumption, WMH white matter hyperintensity. ^*^p < 0.05, ^**^p < 0.01, ^***^p < 0.001.

Similarly, significant indirect effects of age through the pattern’s association with processing speed were observed for the three components of executive functioning—shifting (Effect = 0.062, *SE* = 0.025, 95% CI [0.017; 0.117]), working memory (Effect = −0.033, *SE* = 0.015, 95% CI [−0.067; −0.007]), and inhibition (Effect = −0.040, *SE* = 0.018, 95% CI [−0.080; −0.010]; Fig. 3A-C)—as well as for fine motor function (Effect = 0.021, *SE* = 0.012, 95% CI [0.003; 0.048]; Fig. 4).

Given that GPT can also be considered a measure of processing speed, we additionally tested an alternative model in which the order of the mediators was reversed, such that the relationship between age and processing speed was mediated by the WMH-SGM network pattern and then fine motor function with the same covariates added. This serial mediation model, however, did not reach significance (Effect = 0.021, *SE* = 0.014, 95% CI [−0.001; 0.054]), with no significant association of the network pattern with performance on the GPT (*β* = 0.119, *p* = 0.0518), supporting our initial predicted order of mediators.

The serial mediations followed the same pattern: increasing age was associated with greater expression of the WMH-SGM network pattern, which predicted slower processing speed, which was associated with poorer performance in verbal memory (Fig. 2), executive functioning (Fig. 3), and fine motor function (Fig. 4). The indirect associations between age and cognitive functioning sequentially through the WMH-SGM pattern and processing speed remained significant after additionally adjusting for other vascular health risk factors, including high cholesterol status, smoking history, BMI, and VO_2_max, for verbal memory (Effect = −0.025, *SE* = 0.013, 95% CI [−0.055; −0.005]), shifting (Effect = 0.050, *SE* = 0.023, 95% CI [0.011; 0.099]), working memory (Effect = −0.025, *SE* = 0.013, 95% CI [−0.054; −0.004]), inhibition (Effect = −0.032, *SE* = 0.016, 95% CI [−0.069; −0.006]), and fine motor function (Effect = 0.017, *SE* = 0.011, 95% CI [0.002; 0.043]).

Additionally, further adjustment for depression ratings did not attenuate these indirect effects through processing speed for verbal memory (Effect = −0.026, *SE* = 0.014, 95% CI [−0.058; −0.006]), shifting (Effect = 0.051, *SE* = 0.023, 95% CI [0.011; 0.103]), working memory (Effect = −0.026, *SE* = 0.013, 95% CI [−0.056; −0.004]), inhibition (Effect = −0.033, *SE* = 0.016, 95% CI [−0.070; −0.006]), and fine motor function (Effect = 0.018, *SE* = 0.011, 95% CI [0.003; 0.046]).

## Discussion

Our study investigating the effects of WMH lesions on volumes of subcortical brain structures in cognitively unimpaired, healthy older adults identified a WMH-related regional network covariance pattern of volume reductions in bilateral putamen and left nucleus accumbens with relative volume increases in bilateral caudate nucleus. To understand the pathways linking the pattern of WMH-related SGM volume differences to cognitive aging, we evaluated a series of predicted mediation models and found, along with direct effects of age on cognition, significant indirect effects of the WMH-SGM pattern in the relationship between age and cognition. Specifically, increasing age was associated with greater expression of the WMH-SGM pattern leading to slowed processing speed, which was then sequentially linked to poorer performance in other cognitive domains often impacted by aging. These results suggest that cognitive dysfunction in healthy aging may be, in part, attributable to WMH-related subcortical volumetric differences associated with slowed processing speed.

We identified a subcortical atrophy pattern of volume reductions in putamen and nucleus accumbens associated with global WMH burden in this healthy older adult cohort, suggesting greater sensitivity of volumes of the striatum—putamen and nucleus accumbens—to cerebrovascular risk burden characterized by WMH lesions in the context of healthy aging. These findings are consistent with prior studies that have typically relied on univariate analysis methods showing striatal volume reductions, as well as ventricular enlargement and atrophy of the basal ganglia, in relation to greater WMH volume^9,12–15^. However, our results complement the previous findings by showing WMH-related subcortical volumetric differences as a network covariance pattern of SGM associated with WMH lesion load. These findings help to advance current understanding of the association between WMH lesions and brain atrophy that has been often focused on morphological differences in cortical brain regions or limited to selective individual subcortical brain structures. The atrophy of the putamen and nucleus accumbens may be explained by direct vascular lesions or secondary degeneration^15,39,40^. Due to the surrounding small perforating arteries, these brain structures have been suggested to be particularly vulnerable to ischemic damage associated with small vessel CVD^40^. It has also been suggested that extensive striatal connections with multiple brain regions predispose the putamen and nucleus accumbens to secondary loss of gray matter due to white matter damage, which ultimately can lead to observable atrophy in the involved subcortical structures^15,39,41–43^.

Another main finding of our study is that the WMH-SGM pattern mediated the relationship between age and processing speed, but not memory, executive functioning, and fine motor function, which is consistent with prior research suggesting that processing speed is a cognitive function preferentially affected by WMH burden^2,3,36^. We also found that greater WMH-SGM pattern expression related to age was associated with diminished memory, executive functioning, and fine motor function, but only through slowed processing speed within serial mediation models. These results are in line with previous studies showing poorer performance on tasks of such age-sensitive cognitive domains in relation to greater WMH burden^3–5^ and smaller striatal volume^10,44^. Our findings, however, extend previous findings by demonstrating that WMH-related SGM volume pattern differences in healthy aging contribute to multiple aspects of cognitive dysfunction mainly through its impact on processing speed.

While its involvement in motor control and fine motor skills has long been recognized^42^, the striatum, as part of discrete frontal cortico-striatal networks, has also been reported to contribute to non-motor functions, such as stimulus value representations and related stimulus-driven motivational states, and executive functions (e.g., inhibitory control and task set-shifting)^42,45^. A recent human neuroimaging study has shown that a sub-region of the ventral striatum, including the nucleus accumbens, was associated with effortful task responses, whereas bilateral putamen was engaged in both effortful and non-effortful (or simple) task-related movements^43^.

Moreover, deficits in motivated voluntary behaviors in small vessel CVD have been associated with striatal volume reductions and damage to white matter tracts connecting frontal lobe regions to basal ganglia, particularly involving the ventral striatum^46,47^. These results, taken together, suggest that the relationships between volume reductions of the putamen and nucleus accumbens associated with WMH lesions and slowed processing speed could be related to macrostructural white matter damage by WMH lesions within tracts connecting frontal cortico-striatal brain regions implicated in effort-based motivational performance and associated gray matter volume loss. Further work on how frontal lobe and other cortical brain volumes relate to WMH-related subcortical volumetric differences in the context of healthy aging would be important to help gain a more complete understanding of the impacts of WMH lesions on structural brain and cognitive aging and the risk of dementia.

Consistent with a large body of previous work^37,38^, we observed a mediational influence of processing speed associated with the WMH-SGM pattern on each of three main components of executive functioning (i.e., shifting, working memory, and inhibition) and verbal memory, in addition to age-related direct effects on these cognitive domains indicating age’s unique contribution. These results suggest that some of the age-related decrements in higher-order cognitive functions, across timed and untimed measures, reflect reductions in the speed with which task-relevant cognitive processing operations can be executed. This provides support for the view that diminished processing speed is an important underlying aspect of broader cognitive dysfunction in cross-sectional studies of aging^37,38^.

Furthermore, that significant mediation effects were not observed in the reverse serial mediation model, where the WMH-SGM network pattern and the GPT score sequentially mediated the TMT-A score, supports the directional importance of processing speed in mediating the pattern’s association with fine motor function. Although often considered a test of simple visuomotor speed, the GPT has been suggested to incorporate visuospatial abilities, fine motor control, and manual dexterity^48^, while the TMT-A has been suggested to depend on simple visuomotor tracking abilities^49^. Our results potentially indicate the association of the WMH-SGM pattern with these additional components more specifically concerned with fine motor skills in performance on the GPT, distinct from the TMT-A whose performance may reflect a more basic information processing speed function that serves as a prerequisite for other higher-order cognitive abilities.

It is important to note that our results did not diminish substantially after controlling for common vascular health risk factors, such as hypertension status, high cholesterol status, smoking history, BMI, and cardiorespiratory fitness levels, as well as APOE ε4 status, and further depressed mood. These results suggest the possibility of a more specific vascular health risk process derived from the accumulation of WMH lesions associated with volumetric differences in SGM leading to cognitive dysfunction, which also could not be explained by differences in depressed mood in this healthy older adult cohort. Previous structural neuroimaging studies of non-demented older adults have implicated reduced volumes of putamen and nucleus accumbens in cognitive impairment and increased risk of ADRD, as well as in aging^6–9,11,23,24^. These results from previous studies suggest that WMH-related SGM volume pattern differences leading to cognitive dysfunction may be an important link in the association between small vessel vascular pathology and increased vulnerability to the development of pathological aging leading to dementia^6,8,9^. Examination of the potential role of WMH-related subcortical volumetric differences as an early indicator of dementia risk related to CVD and their potential linkages to the development of Alzheimer’s disease (AD) remains an important direction for future longitudinal work. Additionally, future research on how white matter volume relates to the pattern of WMH-related SGM volume differences and cognition in the context of healthy aging may provide additional insight into structural brain and cognitive aging.

The WMH-SGM network pattern also included covarying areas of relatively increased gray matter in relation to WMH burden in bilateral caudate nucleus. In line with our findings, positive associations of white matter lesions with caudate volume have been reported in non-demented community-dwelling older adults^9^. Such positive associations may reflect perivascular spaces related to age-associated CVD pathology, by which the caudate nucleus has been shown to often be affected due to its anatomical location and specificity^50–52^. It is also possible that the positive associations reflect the presence of periventricular WMH lesions, which can be potentially misclassified as gray matter in the caudate^53^. Further investigations on WMH-related subcortical morphological differences in the context of healthy aging are needed to help clarify the physiological implications of the observed relatively increased gray matter of the caudate with greater WMH burden.

Hippocampal volume has been shown to be associated with global WMH burden in previous studies using univariate analysis^17,18^. We identified a multivariate SSM WMH-SGM pattern focusing on basal ganglia regions without hippocampal volume as a main contributor. The results from studies of cognitively unimpaired or non-demented older adults, however, have been inconsistent, with some studies finding evidence in support of associations between WMH and hippocampal atrophy, but others not^15–18,54,55^. Factors, such as differences in the sample size and composition, study design (e.g., cross-sectional or longitudinal), and analysis methods (e.g., univariate or multivariate), as well as health status of the study samples with a different prevalence of genetic and vascular health risk factors, may help to explain such variation in the findings.

Another factor that may account for some of the discrepancies across studies is differences in the potential influence of WMH spatial distributions and patterns. For instance, hippocampal atrophy has been found in relation to subtypes of WMH, such as deep or periventricular^15^. In addition, a recent study of this healthy older adult cohort found smaller hippocampal volumes associated with increases in left temporal and right parietal lobe WMH volumes^33^. This suggests that hippocampal volume may be preferentially sensitive to a pattern of regional WMH volume differences in healthy aging^33^, which appears distinct from the association of total WMH volume with subcortical brain structures. More research is needed to further evaluate potential differences in specific associations of subtypes and regional differences in WMH volumes with subcortical brain structures in the context of healthy aging and increased risk for CVD and AD dementia.

Our cohort included participants who were generally in good health, well-educated, and predominantly White. Additionally, the relatively small sample size may restrict our ability to detect smaller effects and the generalizability of our findings. As such, it would be important to determine whether our findings are generalizable to more diverse and larger samples of older adults. Although we accounted for key demographic and clinically-relevant characteristics in our analyses, we cannot rule out the possibility that unmeasured factors that could be related to WMH-related subcortical brain atrophy, slowed processing speed, or a combination of these might influence our observed associations. Finally, using mediation modeling with a bootstrapping procedure, we tested a theoretically supported hypothesized pathway linking small vessel CVD to cross-sectional differences in cognitive aging and showed significant, robust indirect effects. The cross-sectional observational study design, however, does not allow for conclusions on causal relationships. Further studies using longitudinal and intervention-based study designs are needed to extend our findings and to clarify whether WMH-related SGM atrophy pattern differences lie on the causal pathway to age-related cognitive impairment.

In conclusion, global WMH burden, in our sample of healthy middle-aged to older adults, was associated with a regionally distributed pattern of SGM atrophy involving the striatum—particularly, volume reductions in the putamen and nucleus accumbens—followed by slowed processing speed and associated decrements in other age-sensitive cognitive domains of verbal memory, executive functions, and fine motor skills. These findings suggest that the multivariate SSM WMH-SGM pattern and its association with processing speed may reflect a potential pathway linking cerebrovascular risk burden due to WMH lesions with broader aspects of cognitive function in healthy older adults. Future research is needed to further investigate how WMH lesion load influences cognitive aging and to assess causality in our observed associations.

## Methods

### Participants

Participants were 178 community-dwelling, healthy older adults (mean age = 69.77 ± 10.22 years, 50.6% women, 95.5% White individuals with 6.2% of the sample self-identifying as Hispanic/Latinx), who completed brain MRI scans, neuropsychological tests, and a graded treadmill exercise test as part of a study on cognitive aging. Participants underwent a neurological examination by a geriatric neurologist (GAH), together with a comprehensive medical screen, to exclude significant medical, psychiatric, and neurological disorders. Exclusion criteria also included: a Mini-Mental State Exam^56^ score < 26 and a Hamilton Depression Rating score^57^ ≥ 10. The sample’s demographic characteristics are reported in Table 2. After being fully informed of the study procedures and possible risks, all participants provided written consent. All procedures for this study were reviewed and approved by the University of Arizona Institutional Review Board and were completed in accordance with institutional guidelines.

**Table 2:** Characteristics of the study sample (*n* = 178)

Variable	*M* (*SD*) or *N* (%)
Age (years)	69.77 (10.22)
Sex (women)	90 (50.6)
Race/ethnicity^a^	
White	170 (95.5)
Asian	3 (1.7)
American Indian/Alaska Native	1 (0.6)
Multiracial^b^	4 (2.2)
Education (years)	16.72 (2.80)
GDS^e^	1.01 (1.58)
MMSE score	29.02 (1.23)
VO_2_max (mL/kg/min)	24.43 (5.81)
BMI (kg/m^2^)	25.21 (3.87)
APOE ε4 carriers	56 (31.5)
Hypertension^c^	59 (33.1)
High cholesterol^d^	125 (70.2)
Smoking history (current or past)	71 (39.9)
Diabetes	7 (3.9)
Total WMH volume (mL)	6.68 (10.28)
SRT CLTR score	63.38 (37.16)
WAIS-IV LNS score	18.77 (2.80)
SCWT score	36.71 (9.33)
TMT-A (seconds)	32.62 (10.67)
TMT-B^e^ (seconds)	75.80 (30.27)
GPT dominant hand^e^ (seconds)	92.10 (26.77)
Time interval^f^ (days)	56.49 (46.69)

*M and SD* represent mean and standard deviation, respectively.

APOE apolipoprotein E, *BMI* body mass index, *CLTR* consistent long-term retrieval, *GDS* Geriatric Depression Scale, *GPT* Grooved Pegboard Test, *LNS* letter number sequencing, *MMSE* Mini-Mental Status Exam, *SCWT* Stroop Color-Word Interference Test, *SRT* Buschke Selective Reminding Test, *TMT* Trail Making Test, *VO*_2_*max* volume of maximum oxygen consumption, *WAIS-IV* Wechsler Adult Intelligence Scale–Fourth Edition, *WMH* white matter hyperintensity.

^a^ Self-reported race and ethnicity.

^b^ Four participants (2.2%) self-reported as multiracial, with two individuals identifying as White and American Indian or Alaska Native, and the other two as White, Black or African American and American Indian or Alaska Native.

^c^ Self-reported history of hypertension.

^d^ Individuals with self-reported history of high cholesterol, use of cholesterol-lowering medications, or total serum cholesterol ≥ 200 mg/dL.

^e^ *n* = 177.

^f^ Time interval between MRI scans and neuropsychological assessment.

### Image acquisition

MRI scans were acquired during a single imaging visit on a 3 T GE Signa scanner (HD Signa Excite, General Electric, Milwaukee, WI). We obtained volumetric T1-weighted images using a spoiled gradient echo sequence (TR = 5.3 ms, TE = 2.0 ms, TI = 500 ms, flip angle = 15°, matrix = 256 × 256, field of view = 25.6 cm, slice thickness = 1.0 mm). We also acquired T2-weighted images using a fluid attenuated inverse recovery (FLAIR) sequence (TR = 11,000 ms, TE = 120 ms, TI = 2,250 ms, flip angle = 90°, matrix = 256 ×256, field of view = 25.0 cm, slice thickness = 2.6 mm).

### Image processing

T1-weighted images were processed using the automated *recon-all* processing stream with standard parameters in FreeSurfer v5.3 (http://surfer.nmr.mgh.harvard.edu) to obtain volumes of the following seven subcortical brain structures for each hemisphere: caudate, putamen, pallidum, hippocampus, nucleus accumbens, amygdala, and thalamus. Details of FreeSurfer’s processing pipeline for subcortical segmentation are described elsewhere^58–60^, which included the following steps: motion correction, intensity normalization, removal of non-brain tissue, segmentation of subcortical gray/white matter volumetric structures, tessellation of gray/white matter and gray matter/pial boundary, Talairach transformation, topology correction, and surface deformation. Outputs of the FreeSurfer segmentation were visually inspected for accuracy, and the *recon-all* pipeline was re-processed, as needed.

WMH volume was segmented automatically from T1 and FLAIR images using the lesion segmentation toolbox (LST)^61^ implemented in Statistical Parametric Mapping (SPM12; Wellcome Trust Center for Neuroimaging, London, UK) and the lesion growth algorithm (LGA). The processing steps for WMH segmentation in this cohort have been previously described in detail^22,62^. Briefly, manually segmented reference WMH maps were generated with ITK-SNAP (www.itksnap.org) in a subset of 35 participants based on a consensus review by a neurologist (GAH) and two expert raters (PKB and GEA). Lesion probability maps were then produced by the LST at the optimization parameter kappa of 0.35, which was determined through assessment of the segmentation accuracy across an array of kappa thresholds (0.05 – 1.00), that was found to have the highest spatial overlap between the reference WMH maps and the LGA-generated WMH maps. Total WMH volume for each participant was computed by summing voxel volumes, and the values were log-transformed for subsequent analyses. TIV was calculated in each participant’s native brain space as the combined volume of segmented gray/white matter and cerebrospinal fluid using T1 scans^63^.

### Cognitive measures

Participants completed a battery of neuropsychological tests that measured cognitive domains typically impacted by aging, including verbal memory, executive functioning, processing speed, and fine motor function^34,35^. Verbal memory was assessed with a 12-word, 12-trial version of the SRT^64^. For the present study, we used the SRT CLTR score as a measure of verbal memory, which reflects the ability to consistently recall items on at least three consecutive trials without reminding^64^.

For executive functioning, we focused on measures of shifting, working memory, and inhibition^65^, which were assessed by time to complete the TMT-B^66^ residualized to remove the influence of processing speed measured by the TMT-A; WAIS-IV LNS subtest score^67^; and SCWT^68^ score, respectively. We used the time to completion on TMT-A^66^ as a measure of processing speed^49^ and the time taken to complete the dominant hand GPT^69^ as a measure of fine motor function^48^ after log-transformation to normalize the distributions for subsequent analyses. One participant had a missing value for the TMT-B, and one participant was missing the GPT.

### Laboratory assessments

APOE alleles were determined on genomic DNA samples by the polymerase chain reaction/restriction enzyme method^70^, as previously described in detail^22^. Briefly, amplified DNA by polymerase chain reaction was digested with HhaI restriction enzyme and analyzed on agarose gel. In this cohort, there were 56 APOE ε4 carriers (31.5%) and 122 ε4 non-carriers (68.5%).

Serum total cholesterol concentrations were measured in fasting blood samples by standardized enzymatic methods in the Clinical Chemistry Laboratory of the Department of Pathology at the University of Arizona Medical Center, Tucson, Arizona, following standard laboratory procedures. For the current study, high cholesterol was defined as self-reported history of high cholesterol, use of cholesterol-lowering medications, or serum total cholesterol ≥ 200 mg/dL^71–73^.

### Cardiorespiratory fitness assessment

Cardiorespiratory fitness, defined as VO_2_max, has been previously described in detail^30^ and was measured with indirect calorimetry during a graded treadmill exercise test using a modified Naughton protocol^74^. Criteria for reaching VO_2_max included (1) a plateau in oxygen consumption in response to increasing workload; (2) a respiratory exchange ratio ≥ 1.1; and (3) a heart rate ± 10 beats/min of the age-predicted maximum^75^. We adjusted mediation models for cardiorespiratory fitness, as well as other common cardiovascular risk factors, to test associations between WMH-related subcortical morphological differences and cognition separate from overall cardiovascular health.

### Statistical analysis

Multivariate SSM analysis^26^ was applied to measures of bilateral volumes of seven SGM structures in Matlab R2020a (MathWorks, Natick, MA) to identify a subcortical regional covariance network pattern related to total WMH volume reflecting the regionally distributed associations of WMH burden with SGM volumes^20,21,30^. With this multivariate analytic approach, regional differences in the WMH-SGM network pattern indicate how the subcortical brain regions covary with each other in relation to WMH volume. In addition, corresponding participant scores are generated, which reflect individual (i.e., between-person) differences in the expression of the network covariance pattern.

As previously described in Alexander and Moeller (1994)^26^, key steps in the application of the SSM include: 1) subtracting mean values across regions and participants after natural log-transformation; 2) applying a modified principal component analysis on the participant by region residual data; 3) obtaining a linearly combined set of SSM components and corresponding participant scores indicating the extent to which the identified WMH-related SGM network pattern was expressed by each participant; 4) using BIC-based multiple linear regressions to identify the set of SSM components whose participant scores best predicted total WMH volume; and 5) performing bootstrap resampling with 10,000 iterations on the point estimate for the WMH-related SGM volume pattern^63,76,77^ to provide CIs for the observed regional pattern weights. We used the BIC^78^ as a model selection approach to achieve balance between model fit and complexity to maximize generalizability of our findings^79^. Regional weights reflect associations between volumes of each subcortical brain structure and WMH lesions in the context of the observed linearly combined SSM network pattern. Negative weights indicate regions showing SGM reductions with greater WMH volumes, whereas positive weights indicate those regions showing WMH-related relative volume increases.

We then tested whether age-related differences in cognitive performance were mediated through the identified WMH-related SGM network pattern in separate simple mediation models. In these models, we focused on one measure for each hypothesized cognitive domain/component to limit Type 1 error across multiple mediation models. The following cognitive measures expected to be related to aging and WMH burden were separately included as an outcome variable^4,30,35,80^—verbal memory (SRT CLTR score); executive functioning (the standardized residual of time to complete TMT-B, WAIS-IV LNS score, and SCWT score); speed of processing (time to complete TMT-A); and fine motor function (time to complete GPT dominant hand).

Given a central role of processing speed in the operation of higher-order cognitive functions^37,38^, these models were extended to test whether speed of processing mediated the pattern’s association with performance in other age-sensitive cognitive domains to further examine how the WMH-SGM pattern contributes to cognitive aging. Specifically, we tested, in separate serial mediation models, age as a predictor and cognitive measures of verbal memory (SRT CLTR score), executive functioning (time to complete TMT-B, WAIS-IV LNS score, and SCWT score), and fine motor function (time to complete GPT dominant hand) as a dependent variable, with the WMH-SGM pattern and processing speed performance on the TMT-A (time to complete TMT-A) evaluated as sequential mediators.

In mediation model analyses, TIV, sex, years of education, APOE ε4 carrier status, hypertension status, and the time interval between administration of the neuropsychological tests and MRI scans were included as covariates to account for their relation to WMH lesions, volumetric reductions in SGM, and cognitive performance^2,16,22,80^. We then repeated the analyses above with smoking history, VO_2_max, BMI, and high cholesterol status as additional covariates to further adjust for the potential influence of other common vascular health risk factors^2^. As a follow-up, depression ratings on the Geriatric Depression Scale^81^ were entered in the serial mediation models as an added covariate to control for potential differences in depressed mood influencing cognitive function.

A bootstrapping procedure was applied to all mediation model analyses with 10,000 iterations to construct 95% percentile CIs for indirect effects using the PROCESS macro v4.2^82^ and SPSS 28.0 (IBM Corp., Armonk, NY). The 95% percentile bootstrap CIs that exclude zero indicate that there are significant mediation effects. Completely standardized indirect effects were computed and reported as measures of effect size^83^. All tests were two-tailed with the alpha level set at 0.05.

## Data Availability

The datasets used during the current study are available from the corresponding author on reasonable request.

## Code availability

Code used for this study is available from the corresponding author on reasonable request.
